# Radical hysterectomy with adjuvant radiotherapy versus radical radiotherapy for FIGO stage IIB cervical cancer

**DOI:** 10.1186/1471-2407-14-63

**Published:** 2014-02-04

**Authors:** Yanlan Chai, Tao Wang, Juan Wang, Yunyi Yang, Ying Gao, Jiyong Gao, Shangfeng Gao, Yueling Wang, Xi Zhou, Zi Liu

**Affiliations:** 1 The Department of Radiotherapy Oncology of the 1st Affiliated Hospital, Xi’an Jiao Tong University, Xi’an 710061, China; 2 The Department of Gynecology of the 1st Affiliated Hospital, Xi’an Jiao Tong University, Xi’an 710061, China; 3 Renmin Hospital, Hubei University of Medicine, Shiyan, Hubei 442000, China

**Keywords:** Cervical carcinoma, Stage IIB, Surgery, Radiotherapy, Adverse effects

## Abstract

**Background:**

The goal of this study was to compare treatment outcomes for Federation of Gynecology and Obstetrics (FIGO) stage IIB cervical carcinoma patients receiving radical surgery followed by adjuvant postoperative radiotherapy versus radical radiotherapy.

**Methods:**

Medical records of FIGO stage IIB cervical cancer patients treated between July 2008 and December 2011 were retrospectively reviewed. A total of 148 patients underwent radical hysterectomy with pelvic lymph node dissection followed by adjuvant radiotherapy (surgery-based group). These patients were compared with 290 patients that received radical radiotherapy alone (RT-based group). Recurrence rates, progression-free survival (PFS), overall survival (OS), local control rates, and treatment-related complications were compared for these two groups.

**Results:**

Similar rates of recurrence (16.89% vs. 12.41%, p = 0.200), PFS (log-rank, p = 0.211), OS (log-rank, p = 0.347), and local control rates (log-rank, p = 0.668) were observed for the surgery-based group and the RT-based group, respectively. Moreover, the incidence of acute grade 3–4 gastrointestinal reactions and late grade 3–4 lower limb lymphedema were significantly higher for the surgery-based group versus the RT-based group. Cox multivariate analyses found no significant difference in survival outcome between the two groups, and tumor diameter and histopathology were identified as significant prognostic factors for OS.

**Conclusions:**

Radical radiotherapy was associated with fewer treatment-related complications and achieved comparable survival outcomes for patients with FIGO stage IIB cervical cancer compared to radical hysterectomy followed by postoperative radiotherapy.

## Background

Cervical cancer is the most common gynecological cancer in developing countries, and Federation of Gynecology and Obstetrics (FIGO) stage IIB cervical cancer is recognized as a locally advanced stage of disease [1]. Currently, there is no international agreement on how FIGO stage IIB patients should be treated. The National Comprehensive Cancer Network (NCCN) guidelines recommend cisplatin-based chemoradiotherapy as a primary treatment for FIGO stage IIB disease [2]. In 2003, the FIGO annual report indicated 72% of patients with FIGO stage IIB cervical cancer received radical radiotherapy [3]. However, other guidelines, such as the German Arbeitsgemeinschaft Gynaekologische Onkologie (AGO) guidelines, recommend radical hysterectomy plus adjuvant radiotherapy as a feasible approach for the treatment of FIGO stage IIB disease.

There are advantages associated with both primary surgery and radical radiotherapy. Primary surgery allows the ovaries to be preserved and avoids early menopause, and may also decrease vaginal fibrosis compared with radical radiotherapy [4,5]. Conversely, radiotherapy can be administered to elderly patients, to patients that have other diseases present, and to patients with extensive vaginal invasion that are predicted to experience severe urinary incontinence following surgery [5,6].

In previous observational studies, the 5-year survival rates for patients with FIGO stage IIB cervical cancer who were treated with radical surgery plus adjuvant radiotherapy were reported to range from 64.0% to 85.2%. This is comparable to the 5-year survival rates of patients treated with definitive radiotherapy (64.0–81.1%) [1,3,7,8]. However, treatment-related complications, which can significantly affect a patient’s quality of life, are a critical consideration when deciding between two treatment modalities with equivalent survival outcomes [9]. As reported, combined treatment of radical hysterectomy plus adjuvant radiotherapy is associated with a significantly higher rate of morbidity compared with surgical treatment alone [10]. Thus, it has been suggested that radical radiotherapy is beneficial for FIGO stage IIB cervical cancer patients who require adjuvant radiotherapy following radical hysterectomy [10].

To our knowledge, a comparison of radical surgery followed by adjuvant postoperative radiotherapy versus radical radiotherapy for patients with stage IIB cervical cancer in China has not been reported. Therefore, in this retrospective study, survival outcomes and treatment-related complications associated with FIGO stage IIB cervical cancer patients from the Chinese population who underwent radical hysterectomy plus postoperative radiotherapy versus radical radiotherapy were compared and evaluated.

## Methods

### Patients

Data acquisition and the analysis of medical records for 438 patients with stage IIB primary cervical cancer who were treated at The First Affiliated Hospital of Xi’an Jiaotong University between July 2008 and December 2011 were approved by the Ethics Committee of The First Affiliated Hospital of Xi’an Jiaotong University. The following inclusion criteria were used for this study: a histological diagnosis of FIGO stage IIB disease; an absence of prior treatment; a Karnofsky Performance Status ≥ 80; and the completion of (a) primary surgery consisting of radical hysterectomy with pelvic lymphadenectomy combined with adjuvant radiotherapy, or (b) radical radiotherapy with concurrent chemotherapy. A pelvic examination in the absence of anesthesia was conducted, to evaluate patients according to the FIGO staging system. Baseline data were available from computed tomography (CT) of the chest and abdomen, magnetic resonance imaging (MRI) of the pelvis, and complete blood count and biochemistry panels. Furthermore, intravenous pyelography, cystoscopy, and sigmoidoscopy were considered optional. Lymph nodes measuring 1 cm or greater across their largest diameter on CT or MRI scans were defined as metastatic nodes.

### Treatment

All of the patients in the surgery-based group underwent radical hysterectomy with pelvic lymph node dissection by laparotomy. Radical hysterectomy included resection of the uterus along with its attached parametrial soft tissue and a margin of the upper vagina. The lymphadenectomy procedure included a complete bilateral pelvic lymphadenectomy intending to remove all of the external iliac, internal iliac, common iliac, obturator, suprainguinal, and presacral lymph nodes. External beam radiotherapy (EBRT) was delivered 2–4 weeks later, using a linear accelerator of three-dimensional conformal radiation therapy (3D-CRT). CT-based treatment planning was used for all patients. According to Radiation Therapy Oncology Group (RTOG) guidelines [11], the clinical target volume (CTV) included the common, external, and internal iliac lymph node regions and the upper 3.0 cm of the vagina. The superior margin of the external radiation field was located at the abdominal aortic bifurcation, and the inferior border extended 3.0 cm below the upper extent of the vagina (defined by the vaginal marker), or to 1.0 cm above the inferior extent of the obturator foramen. External irradiation was delivered to the whole pelvis (1.8 Gy or 2 Gy per fraction), with five fractions administered per week for a total of 25 fractions and 45–50 Gy. If common iliac lymph node metastasis was detected, extended field radiotherapy was additionally administered. For patients that displayed vaginal invasion close to the surgical margin (≤ 0.5 cm), they received intracavitary radiotherapy (ICRT). Patients with one or more pathologic risk factors (e.g., positive nodes, positive surgical margin) were administered paclitaxel (135 mg/m^2^ for the first day) and cisplatin (25 mg/m2 per day for 3 consecutive days) (TP regimen) every 21–28 d for four cycles. All patients received antiemetic drugs and were pretreated with corticosteroids, diphenhydramine, and H2 antagonists.

Radical radiotherapy consisted of pelvic EBRT followed by high dose rate intracavitary brachytherapy (HDR-ICBT). Pelvic EBRT was administered using a linear accelerator of 3D-CRT. The pelvic radiation field was the same as that employed for adjuvant radiotherapy, yet it was extended inferiorly in cases involving vaginal invasion. External irradiation was delivered to the whole pelvis (1.8–2 Gy per fraction), with five fractions administered per week for a total of 25–28 fractions and 50–50.4 Gy. After completing external irradiation, gynecological examinations and pelvic MRI were performed to determine the appropriate ICBT program and dose. ICBT was performed using the Fletcher-Suit-Delclos set with a microSelectron HDR (Nucletron, Veenendaal, Netherlands). Loading was based on the Manchester radium system. Orthogonal x-rays were administered after each insertion in order to calculate the dwell times for the prescribed Point A dose. Point A was defined as 2 cm above the cervical os marker and 2 cm perpendicular to the uterine axis along the plane of the uterus. Vaginal packing was used to maximize the distances from the source to the bladder wall and the rectal wall. The total planned dose to point A for HDR-ICBT was 24–25 Gy, and it was administered in four or five fractions. All of the patients in the RT group received one cycle of cisplatin (25 mg/m2 per day for 3 consecutive days) combined with 5-fluorouracil (300 g/m2 per day for 5 consecutive days) (PF regimen) during pelvic EBRT as a radiosensitizing agent. In addition, all patients were administered antiemetic drugs prior to chemotherapy.

### Patient follow-up

Upon completion of treatment, patients were evaluated every three months for the first year, every six months the second through fifth years, and annually thereafter. Gynecologic examination and supraclavicular lymph node palpation were performed at each appointment. Chest x-rays were obtained one year after treatment. Suspected cases of persistent or recurrent disease were confirmed by biopsy whenever possible. For these cases, chest CT and abdomino-pelvic CT or MRI were obtained to detect the site of failure. Patient follow-up was maintained through July 31, 2013. The median duration of follow-up was 36 months (range: 12–60 months) for the surgery-based group and 39 months (range: 16–60 months) for the RT-based group.

### Definition of outcomes and toxicity

Progression-free survival (PFS) was defined as the time interval between the initial diagnosis (at initial biopsy) and the detection of recurrence or death from any cause. Overall survival (OS) was defined as the time from initial diagnosis until death by cervical cancer. Surviving patients and patients with an unknown date of death were censored on the date of last follow-up. Rates of local recurrence (confined to the pelvis) and distant metastasis (any site) were also reported for each patient.

Adverse effects that occurred within 90 days from the start of primary treatment were considered acute complications, and those that occurred 90 days or later from the start of treatment were considered late complications. The severity of the complications associated with radiotherapy or chemotherapy were classified according to the National Cancer Institute Common Toxicity Criteria (NCI-CTC v2.0) and the Radiation Therapy Oncology Group/European Organization for Research and Treatment of Cancer (RTOG/EORTC) criteria, respectively.

### Statistical analysis

Differences between the two treatment groups were assessed using a *χ*^2^ test or Fisher’s exact test for categorical variables. An independent sample *t*-test was used for continuous variables. Survival curves were obtained using the Kaplan-Meier method and were compared using nonparametric survival analysis (log-rank test). A P-value less than 0.05 were considered statistically significant. Multivariate analysis was performed using the Cox proportional-hazard model with a stepwise method (forward selection). P-values less than 0.05 were used as inclusion criteria and P-values greater than 0.10 were used as exclusion criteria. Statistical Package for Social Scientists (SPSS, version 18.0, IL) was used for all analyses.

## Results

### Patient characteristics

Of the 438 patients who were eligible for a retrospective analysis of their cases, 148 were treated with radical hysterectomy combined with adjuvant chemoradiotherapy or radiotherapy according to their pathological risk factors (surgery-based group). In addition, 290 patients were treated with radical radiotherapy (RT-based group) (Table 1).

**Table 1:** Patient characteristics

**Characteristic**	**Surgery-based group**	**RT-based group**	***P*-value**
Total no. patients	148	290	
Median age, y (range)	48 (25–70)	51 (24–88)	0.003
Histopathology			0.456
Squamous cell carcinoma	136 (92.0%)	272 (93.8%)	
Non-Squamous cell carcinoma	12 (8.0%)	18 (6.2%)	
Pelvic nodal status (Radiological evidence)			0.356
Positive	27 (18.24%)	43 (14.83%)	
Negative	121 (81.76%)	247 (85.17%)	
Maximum tumor diameter (mm)			0.003
> 40	26 (17.57%)	89 (30.69%)	
≤ 40	122 (82.43%)	201 (69.31%)	
Pretreatment hemoglobin level (g/L)	112.12 ± 10.53	113.56 ± 8.41	0.140

The median ages for the surgery-based and RT-based groups were 48 years (range, 25–70) and 51 years (range, 24–88), respectively, and this difference was significant (P = 0.003). Twenty-six (17.57%) patients in the surgery-based group and 89 (30.69%) patients in the RT-based group had a maximum tumor diameter > 4 cm, and this difference was also significant (P = 0.003). In contrast, pelvic nodal status, histopathology distribution, and pretreatment hemoglobin levels did not significantly differ between the two treatment groups.

In the surgery-based group, 69 patients (46.62%) had one or more risk factors and received TP regimen for four cycles. Of these patients, 59 (39.86%) had histologically positive pelvic nodes, 3 (2.03%) of which had common iliac lymph node metastasis and received additional extended field radiotherapy, while 10 patients (6.76%) had positive vaginal margins and received ICRT. All patients in the RT-based group were administered one cycle of PF regimen.

### Treatment outcome

The follow-up time for the two groups did not significantly differ (P > 0.05). During the follow-up period, 25 (16.89%) patients in the surgery-based group and 36 (12.41%) patients in the RT-based group experienced tumor recurrence (Table 2). The former included recurrence in the pelvis (n = 6, 4.05%), in distant areas outside of the pelvis (n = 17, 11.49%), and in both pelvic and distant areas (n = 2, 1.35%). The same regions for the latter group involved 15 (5.17%), 20 (6.89%), and 1 (0.30%) cases, respectively. The recurrence rate and pattern of recurrence did not significantly differ between the two treatment groups (P = 0.200 and P = 0.224, respectively). Moreover, at the time of this report, 20 (13.51%) patients in the surgery-based group and 29 (10.00%) patients in the RT-based group had died of cervical cancer. One (0.34%) patient from the RT-based group also died due to other causes. Mortality rates for the two treatment groups did not significantly differ (P = 0.324).

**Table 2:** Treatment outcome

**Characteristic**	**Surgery-based group**	**RT-based group**	***P*-value**
Total no. patients	148	290	
Patients with recurrence, N (%)	25 (16.89%)	36 (12.41%)	0.200
Site of recurrence			0.224
Pelvis	6 (4.05%)	15 (5.17%)	
Pelvis plus distant	2 (1.35%)	1 (0.30%)	
Distant	17 (11.49%)	20 (6.89%)	
Total deaths			0.324
N (%)	20 (13.51%)	30 (10.34%)	
Disease	20 (13.51%)	29 (10.0%)	
Other	0 (0%)	1 (0.34%)	

**Figure 1:**
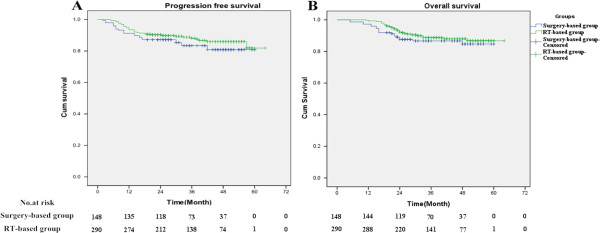
**Kaplan-Meier PFS (A) and OS survival (B) curves for the surgery-based group and RT-based group.** Both rates were similar for the two groups (log-rank, P = 0.211 and P = 0.347, respectively).

**Figure 2:**
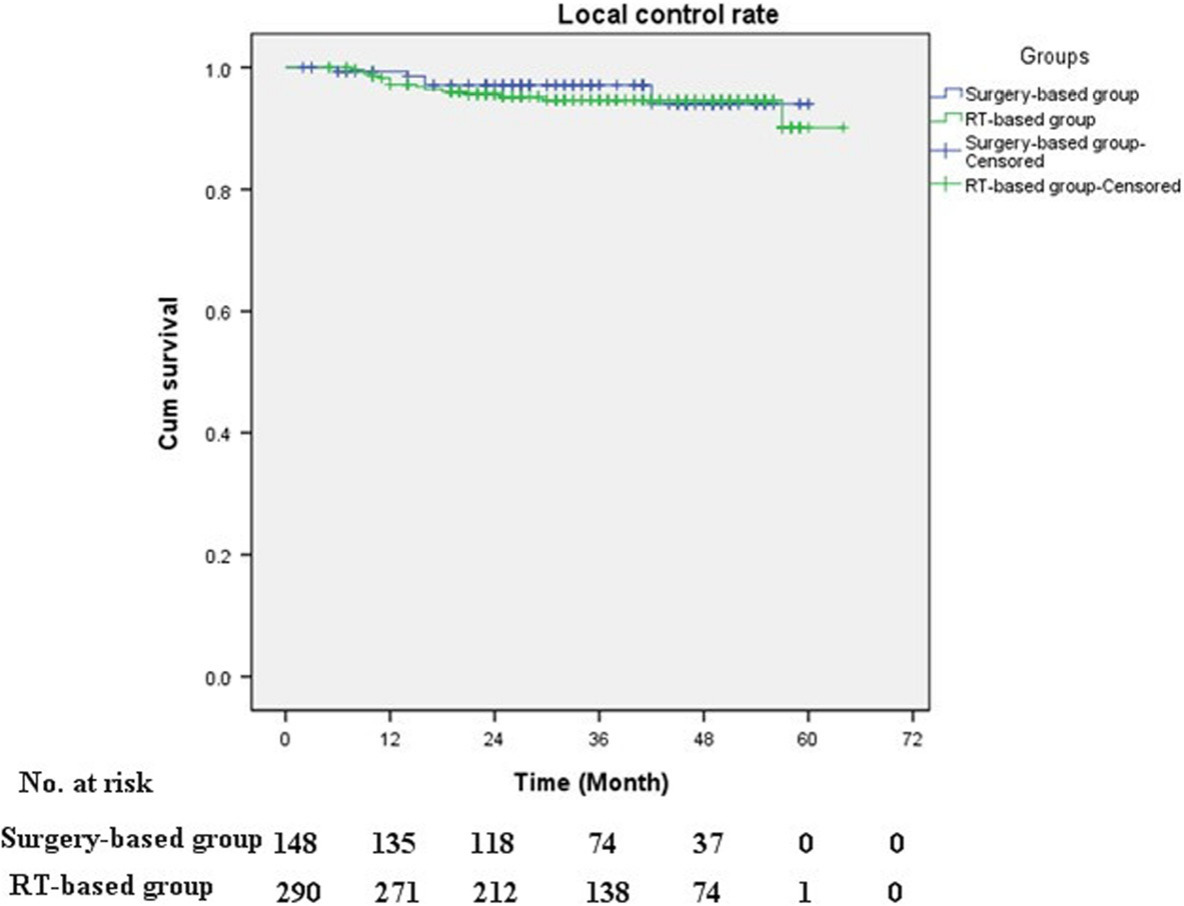
**Cumulative pelvic recurrence rates were calculated for patients in the surgery-based group versus the RT-based group.** These rates were similar for the two groups (log-rank, P = 0.668).

Although the RT-based group exhibited slightly higher PFS rates and OS rates (Figure 1), the differences were not statistically significant (log-rank; P = 0.211 and P = 0.347, respectively). The 5-year PFS rates were 80.8% for the surgery-based group and 86.0% for the RT-based group, while the estimated 5-year OS rates were 84.7% and 86.8%, respectively. The pelvic recurrence rate was similar for the patients in the surgery-based group and those in the RT-based group (Figure 2, log-rank, P = 0.668).

After controlling for other variables in the multivariate analysis, no significant difference in survival outcome was detected between the two treatment groups (Table 3). However, multivariate analysis did demonstrate that histology and tumor diameter were significant prognostic factors for OS.

**Table 3:** Multivariate analysis for survival outcome

**Characteristic**	**PFS**		**OS**	
	**HR (95% CI)**	**P-value**	**HR (95% CI)**	**P-value**
Age, y (> 45 vs. ≤ 45)	0.774 (0.454–1.318)	0.345	0.986 (0.542–1.796)	0.964
Maximum tumor diameter	1.379	0.264	2.023	0.021
(> 40 mm vs. ≤ 40 mm)	(0.785–2.422)		(1.114–3.672)	
Histology	0.832	0.695	0.440	0.045
(SCC vs. non-SCC)	(0.331–2.087)		(0.197–0.982)	
Pre-RT hemoglobin (g/L)	0.654	0.100	0.788	0.406
(> 110 vs. ≤ 110)	(0.394–1.084)		(0.449–1.383)	
Treatment				
Surgery + adjuvant RT	1.441	0.170	1.466	0.195
vs. radical RT	(0.856–2.428)		(0.822–2.615)	

PFS: progression-free survival; OS: overall survival; HR: hazard ratio, 95% CI: 95% confidence interval, SCC: squamous cell carcinoma, RT: radiotherapy.

### Adverse effects

No treatment-related deaths were reported for the cohort examined. Surgery-related complications were classified according to the Clavien-Dindo guidelines [12]. For 148 patients in the surgery-based group, 90 (60.81%) did not experience surgery-related complications. However, grade I complications were reported for 5 (3.38%) patients, and these mainly included fever and pain. Grade II complications were reported for 47 (31.76%) patients, and these included wound infections (n = 5) and bladder dysfunction (n = 42). Furthermore, 2 (1.35%) patients had large infected lymphocysts (grade IIIa) and 4 (2.70%) patient suffered a ureteral fistula (grade IIIb). All of these complications recovered within four weeks.

As shown in Table 4, the frequencies of acute grade 3–4 neutropenia and anemia were similar for the two reatment groups (P = 0.610 and P = 0.067, respectively). For the 148 patients in the surgery-based group, 15 (10.14%) patients suffered grade 3–4 acute gastrointestinal reactions, with 3 (2.03%) patients developing small bowel obstruction (SBO) and 12 (8.11%) patients having diarrhea, both of which were treated conservatively. Among the 290 patients treated with radical radiotherapy, 13 (4.48%) patients suffered diarrhea and were treated conservatively. Overall, the incidence of acute grade 3–4 gastrointestinal reactions was significantly higher for the surgery-based group compared to the RT-based group (P = 0.036). It is possible that this was due to inflammation in the pelvis, intestinal wall edema, and gastrointestinal irritation that developed as a result of the surgery performed.

**Table 4:** Grade 3–4 acute and late stage toxicities

**Characteristic**	**Surgery-based group**	**RT-based group**	***P*-value**
Total no. patients	148	290	
Grade 3–4 acute toxicity			
Neutropenia	50 (33.8%)	91 (31.4%)	0.610
Anemia	12 (8.11%)	41 (14.1%)	0.067
Gastrointestinal reactions	15 (10.1%)	13 (4.48%)	0.036
Small bowel obstruction	3 (2.03%)	0 (0.00%)	
Diarrhea	12 (8.11%)	13 (4.48%)	
Grade 3–4 late stage toxicity			
Chronic radiation intestinal injury	11 (7.43%)	12 (4.14%)	0.144
Small bowel obstruction	2 (1.35%)	0 (0.00%)	
Proctosigmoiditis	9 (6.08%)	12 (4.14%)	
Chronic radiation cystitis	6 (4.05%)	7 (2.41%)	0.378
Lower limb lymphedema	10 (6.76%)	6 (2.07%)	0.017

Radiation enteritis, cystitis, and lower limb edema were the most common late stage toxicities reported. In the surgery-based group, 11 (7.43%) patients developed chronic radiation intestinal injury, with 2 (1.35%) patients developing SBO (one was treated with enterolysis and one was treated conservatively) and 9 (6.08%) patients experiencing proctosigmoiditis. The latter was alleviated by adjusting the patients’ diets and administering Chinese medicine. The same treatment was used for the 12 (4.14%) in the RT-based group that also developed chronic proctosigmoiditis. In addition, chronic radiation cystitis was reported for 6 (10.81%) patients in the surgery-based group and 7 (3.45%) patients in the RT-based group. When the two groups were compared, the frequencies of late stage grade 3–4 radiation intestinal injury and cystitis were similar (P = 0.144 and P = 0.378, respectively). Ten (6.76%) patients in the surgery-based group and 6 (2.07%) patients in the RT-based group suffered severe lower limb edema, respectively, and this difference was significant (P = 0.017) (Table 4). Lymph reflux disorder may account for this phenomenon as a result of the lymph node dissections that were performed.

## Discussion

In the present study, 438 patients with FIGO stage IIB cervical cancer treated with radical surgery plus adjuvant radiotherapy (n =148) or radical radiotherapy (n = 290) were retrospectively analyzed. OS and PFS did not significantly differ for the two treatment groups, although the RT-based group showed slightly higher PFS and OS rates (log-rank, P = 0.211 and P = 0.347, respectively). Previously, Yamashita et al. [7] retrospectively compared the survival of stage IIB patients who underwent radical hysterectomy followed by adjuvant radiotherapy versus those who underwent radiotherapy. The reported 5-year cause-specific survival rates for the two groups were 81.1% and 81.2%, respectively. Furthermore, the difference was not statistically significant. Ohara et al. [8] reported 5-year cause-specific survival rates of 70.5% and 85.2% for the radiotherapy group and the radical hysterectomy group of FIGO stage IIB cervical cancer patients, respectively, while Takahiro et al. [1] reported 5-year survival rates of 69% for both a radical hysterectomy group and a radiotherapy group. Therefore, the results of the present study are consistent with previous findings [1,8,11,13].

In general, the complications associated with radical hysterectomy with adjuvant radiation therapy or radical radiotherapy can include proctitis, cystitis, lower limb lymphedema, urinary and rectal fistula, rectal stricture, and bladder dysfunction [14,15]. In the current study, radiation enteritis, cystitis and lower limb edema were the most common late stage toxicities reported. These conditions typically developed in the first two years following treatment, and were largely alleviated by diet adjustments and Chinese medicine. However, treatment of lower limb edema remains a challenge. The frequency of late lower limb lymphedema for the surgery-based group was significantly higher than that observed for the RT-based group. It is important that patients are informed of the lifelong risks of these complications prior to surgery, since these adverse reactions can lead to a serious decline in patient quality of life. Moreover, careful post-treatment follow-up and timely treatment of complications can help avoid more serious adverse complications and reduce the need for major interventions.

Considering the relatively high incidence of severe complications observed in both treatment groups in the current study, further efforts need to be made to reduce the incidences of these complications. One possibility is the use of more conformal dose distributions with intensity-modulated radiation therapy (IMRT) [16]. There have been a considerable number of studies that have demonstrated that IMRT improves dose distributions and is associated with reduced rates of toxicities, while providing comparable clinical outcomes [17,18]. It will be important for additional clinical trials of IMRT to be conducted to evaluate adjuvant and radical radiotherapy for stage IIB cervical disease.

Patients with FIGO stage IIB cervical cancer usually exhibit high risk pathological factors following radical hysterectomy such as positive pelvic nodes, parametrial invasion, or a positive surgical margin. For these individuals, postoperative adjuvant radiotherapy is inevitable. Consequently, opting for radical radiotherapy instead of radical surgery, especially for patients exhibiting risk factors for the development of severe late stage toxicities [19,20], may provide a better quality of life.

In recent literature, the use of neoadjuvant chemotherapy (NACT) followed by radical surgery for patients with FIGO stage IIB cervical cancer has shown an increasing trend. Both Gadducci et al. [21] and Minig et al. [22] found that neoadjuvant chemotherapy followed by radical surgery was also an effective therapeutic option for patients with FIGO stage IIB cervical cancer. Moreover, this approach had comparable 5-year PFS and OS rates compared to radical radiotherapy. At present, a number of clinical trials involving the application of NACT for patients with FIGO stage IIB cervical cancer are ongoing. When these results are released, the advantages and disadvantages of NACT for this population can be further examined.

In the current study, tumor diameter and histopathology were identified as significant prognostic factors for OS (Table 3). These results are consistent with those of previous studies where tumor size [23,24] and non-squamous histological type [24-26] were identified as prognostic factors for the survival of FIGO stage IIB cervical cancer patients. Thus, the development of new treatment strategies for patients with known risk factors for survival is urgently needed. For this purpose, novel treatments that include the use of new cytotoxic and/or biologic agents as radiosensitizers, or the addition of consolidation chemotherapy following postoperative adjuvant radiotherapy or definitive radiotherapy, should be investigated in future clinical trials [7].

Regarding the limitations of the present study, its retrospective nature is a key aspect. Potential confounding biases may also have been missed in the analyses performed, such as the selection bias introduced by physicians in determining which patients should be considered for radical surgery plus adjuvant radiotherapy versus radical radiotherapy alone. Patients in the surgery-based group were significantly younger and typically had tumors with smaller diameters, indicating that a greater number of patients with favorable prognoses were allocated to the surgery-based group. There are several possible explanations for this. Surgery is preferred for the treatment of younger cervical cancer patients. In addition, surgeons prefer surgical treatment for patients with smaller tumors since the surgery is relatively straightforward. Another limitation of the present study is the imbalanced number of patients in each treatment group and the short follow-up period. A prospective, randomized controlled study would eliminate these biases.

## Conclusion

In conclusion, radical radiotherapy was found to be a safer treatment approach compared to radical hysterectomy followed by postoperative radiotherapy for FIGO stage IIB cervical cancer. Specifically, radical radiotherapy was associated with fewer treatment-related complications and achieved a comparable survival outcome. To confirm the superiority of radical radiotherapy for FIGO stage IIB cervical cancer, survival outcome, frequency of treatment-related complications, and patient quality of life following radical radiotherapy versus radical surgery followed by adjuvant postoperative radiotherapy needs to be evaluated in a randomized controlled trial.

## Consent

Written informed consent was obtained from the patient for the publication of this report and any accompanying images.

## Abbreviations

FIGO: Federation of gynecology and obstetrics; PFS: Progression-free survival; OS: Overall survival; NCCN: National comprehensive cancer network; CT: Computed tomography; MRI: Magnetic resonance imaging; EBRT: External beam radiotherapy; 3D-CRT: Three-dimensional conformal radiation therapy; RTOG: Radiation therapy oncology group; CTV: Clinical target volume; ICRT: Intracavitary radiotherapy; HDR-ICBT: High dose rate intracavitary brachytherapy; RIAISs: Radiation-induced acute intestinal symptoms; IMRT: Intensity-modulated radiation therapy; NACT: Neoadjuvant chemotherapy.

## Competing interest

The authors declare that they have no competing interests. This paper has not been published previously. This study will not be published elsewhere in the same form, in English or in any other language, without consent of the publisher.

## Authors’ contributions

YC guarantees the integrity of the entire study and drafted the manuscript. JW and YG collected medical records. TW and YY performed statistical analyses. YG, JG, and SG searched and arranged the references. YW and XZ modified the manuscript. ZL conceived the study, participated in its design and coordination, and completed the final proofreading of the manuscript. All authors have read and approve the final manuscript.

## Pre-publication history

The pre-publication history for this paper can be accessed here:

http://www.biomedcentral.com/1471-2407/14/63/prepub

## References

[B1] Kasamatsu T Onda T Sawada M Kato T Ikeda S Radical hysterectomy for FIGO stage IIB cervical cancer: clinicopathological characteristics and prognostic evaluation Gynecol Oncol 2009 114 69 148 10.1016/j.ygyno.2009.03.026 19398126

[B2] NCCN Practice Guidelines in Oncology 2013 http://www.nccn.org/professionals/physician_gls/f_guidelines.asp

[B3] Benedet JL Odicino F Maisonneuve P Beller U Creasman WT Heintz AP Ngan HY Pecorelli S Carcinoma of the cervix uteri Int J Gynaecol Obstet 2003 83 Suppl 1 41 78 14763169 10.1016/s0020-7292(03)90115-9

[B4] Landoni F Maneo A Colombo A Placa F Milani R Perego P Favini G Ferri L Mangioni C Randomised study of radical surgery versus radiotherapy for stage Ib–IIa cervical cancer Lancet 1997 350 535 540 10.1016/S0140-6736(97)02250-2 9284774

[B5] Undurraga M Loubeyre P Dubuisson JB Schneider D Petignat P Early-stage cervical cancer: is surgery better than radiotherapy? Expert Rev Anticancer Ther 2010 10 451 460 10.1586/era.09.192 20214525

[B6] Suprasert P Srisomboon J Kasamatsu T Radical hysterectomy for stage IIB cervical cancer: a review Int J Gynecol Cancer 2005 15 995 1001 10.1111/j.1525-1438.2005.00259.x 16343175

[B7] Yamashita H Nakagawa K Tago M Shiraishi K Nakamura N Ohtomo K Oda K Nakagawa S Yasugi T Taketani Y Comparison between conventional surgery and radiotherapy for FIGO stage I–II cervical carcinoma: a retrospective Japanese study Gynecol Oncol 2005 97 834 839 10.1016/j.ygyno.2005.03.017 15894366

[B8] Ohara K Sugahara S Kagei K Hata M Igaki H Tokuuye K Akine Y Retrospective comparison of clinical outcome between radiotherapy alone and surgery plus postoperative radiotherapy in the treatment of stages IB–IIB cervical squamous cell carcinoma Radiat Med 2004 22 42 48 15053175

[B9] Mabuchi S Okazawa M Isohashi F Matsuo K Ohta Y Suzuki O Yoshioka Y Enomoto T Kamiura S Kimura T Radical hysterectomy with adjuvant radiotherapy versus definitive radiotherapy alone for FIGO stage IIB cervical cancer Gynecol Oncol 2011 123 2 241 247 10.1016/j.ygyno.2011.07.009 21820708

[B10] Rotman M Sedlis A Piedmonte MR Bundy B Lentz SS Muderspach LI Zaino RJ A phase III randomized trial of postoperative pelvic irradiation in Stage IB cervical carcinoma with poor prognostic features: follow-up of a gynecologic oncology group study Int J Radiat Oncol Biol Phys 2006 65 1 169 176 10.1016/j.ijrobp.2005.10.019 16427212

[B11] Small W Jr Mell LK Anderson P Creutzberg C De Los SJ Gaffney D Jhingran A Portelance L Schefter T Iyer R Varia M Winter K Mundt AJ Consensus guidelines for delineation of clinical target volume for Intensity-Modulated pelvic radiotherapy in postoperative treatment of endometrial and cervical cancer Int J Radiat Oncol Biol Phys 2008 71 2 428 434 10.1016/j.ijrobp.2007.09.042 18037584 PMC2752724

[B12] Oranusi CK Nwofor A Oranusi IO Complication rates of open transvesical prostatectomy according to the Clavien–Dindo classification system Niger J Clin Pract 2012 15 1 34 37 10.4103/1119-3077.94094 22437086

[B13] Park TK Kwon JY Kim SW Kim SH Kim SN Kim GE Patterns of treatment failure following radiotherapy with combination chemotherapy for patients with high-riskstage IIB cervical carcinoma Int J Clin Oncol 2004 9 2 120 124 10.1007/s10147-003-0378-9 15108044

[B14] Einstein MH Rash JK Chappell RJ Swietlik JM Hollenberg JP Connor JP Quality of life in cervical cancer survivors: patient and provider perspectives on common complications of cervical cancer and treatment Gynecol Oncol 2012 125 1 163 167 10.1016/j.ygyno.2011.10.033 22063460

[B15] Hsu WC Chung NN Chen YC Ting LL Wang PM Hsieh PC Chan SC Comparison of surgery or radiotherapy on complications and quality of life in patients with the stage IB and IIA uterine cervical cancer Gynecol Oncol 2009 115 1 41 45 10.1016/j.ygyno.2009.06.028 19615724

[B16] Hasselle MD Rose BS Kochanski JD Nath SK Bafana R Yashar CM Hasan Y Roeske JC Mundt AJ Mell LK Clinical outcomes of intensity-modulated pelvic radiation therapy for carcinoma of the cervix Int J Radiat Oncol Biol Phys 2011 80 1436 1445 10.1016/j.ijrobp.2010.04.041 20708346

[B17] Folkert MR Shih KK Abu-Rustum NR Jewell E Kollmeier MA Makker V Barakat RR Alektiar KM Postoperative pelvic intensity-modulated radiotherapy and concurrent chemotherapy in intermediate- and high-risk cervical cancer Gynecol Oncol 2013 128 2 288 293 10.1016/j.ygyno.2012.11.012 23159818

[B18] Du XL Tao J Sheng XG Lu CH Yu H Wang C Song QQ Li QS Pan CX Intensity-modulated radiation therapy for advanced cervical cancer: a comparison of dosimetric and clinical outcomes with conventional radiotherapy Gynecol Oncol 2012 125 1 151 157 10.1016/j.ygyno.2011.12.432 22198339

[B19] Chen SW Liang JA Yang SN Liu RT Lin FJ The prediction of late rectal complications following the treatment of uterine cervical cancer by high-dose-rate brachytherapy Int J Radiat Oncol Biol Phys 2000 47 4 955 961 10.1016/S0360-3016(00)00559-9 10863065

[B20] Gondi V Bentzen SM Sklenar KL Dunn EF Petereit DG Tannehill SP Straub M Bradley KA Severe late toxicities following concomitant chemoradiotherapy compared to radiotherapy alone in cervical cancer: an inter-era analysis Int J Radiat Oncol Biol Phys 2012 84 4 973 982 10.1016/j.ijrobp.2012.01.064 22898381 PMC3706199

[B21] Gadducci A Sartori E Maggino T Zola P Cosio S Zizioli V Lapresa M Piovano E Landoni F Pathological response on surgical samples is an independent prognostic variable for patients with Stage Ib2-IIb cervical cancer treated with neoadjuvant chemotherapy and radical hysterectomy: An Italian multicenterretrospective study (CTF Study) Gynecol Oncol 2013 3 pii: S0090-8258(13)01203-1 10.1016/j.ygyno.2013.09.029 24096111

[B22] Minig L Colombo N Zanagnolo V Landoni F Bocciolone L Cárdenas-Rebollo JM Iodice S Maggioni A Platinum-based neoadjuvant chemotherapy followed by radical surgery for cervical carcinoma international federation of gynecology and obstetrics stage IB2-IIB Int J Gynecol Cancer 2013 [Epub ahead of print] 10.1097/IGC.0b013e3182a616d2 24100590

[B23] Horn LC Fischer U Raptis G Bilek K Hentschel B Tumor size is of prognostic value in surgically treated FIGO stage II cervical cancer Gynecol Oncol 2007 107 310 315 10.1016/j.ygyno.2007.06.026 17826822

[B24] Reig A Membrive I Foro P Sanz X Rodríguez N Lozano J Lacruz M Quera J Fernández-Velilla E Algara M Long-term results and prognostic factors of patients with cervical carcinoma treated with concurrent chemoradiotherapy Clin Transl Oncol 2011 13 7 504 508 10.1007/s12094-011-0688-8 21775278

[B25] Xie XZ Song K Cui B Jiang J Zhang YZ Wang B Yang XS Kong BH Clinical and pathological factors related to the prognosis of Chinese patients with stage Ib to IIb cervical cancer Asian Pac J Cancer Prev 2012 13 11 5505 5510 10.7314/APJCP.2012.13.11.5505 23317208

[B26] Galic V Herzog TJ Lewin SN Neugut AI Burke WM Lu YS Hershman DL Wright JD Prognostic significance of adenocarcinoma histology in women with cervical cancer Gynecol Oncol 2012 125 2 287 291 10.1016/j.ygyno.2012.01.012 22266551

